# Electrostatically Tuned Optical Filters Based on Hybrid Plasmonic-Dielectric Thin Films for Hyperspectral Imaging

**DOI:** 10.3390/mi12070767

**Published:** 2021-06-29

**Authors:** Ahmed Abdelghfar, Mohamed A. Mousa, Bassant M. Fouad, Ahmed H. Saad, Noha Anous, Noha Gaber

**Affiliations:** 1 Center for Nanotechnology, Zewail City of Science and Technology, October Gardens, Giza 12578, Egypt; s-mohamedashraf0@zewailcity.edu.eg (M.A.M.); s-bassant_mohamed@zewailcity.edu.eg (B.M.F.); s-ahmedhashem@zewailcity.edu.eg (A.H.S.); 2 Faculty of Engineering, Ain-Shams University, 1 Elsarayat St., Abbassia, Cairo 11517, Egypt; noha.anous@gmail.com

**Keywords:** tunable optical filter, visible range filters, hyperspectral imaging, plasmonic filters, MEMS tunable filters, electrostatic actuators

## Abstract

Hyperspectral imaging has a wide range of uses, from medical diagnostics to crop monitoring; however, conventional hyperspectral imaging systems are relatively slow, bulky, and rather costly. In this paper, we present an inexpensive, compact tunable optical filter for hyperspectral applications. The filter is based on a Fabry-Pérot interferometer utilizing hybrid metallic-dielectric mirrors and actuated using a MEMS electrostatic actuator. The optical filter is designed using the transfer matrix method; then, the results were verified by an electromagnetic wave simulator. The actuator is based on a ring-shaped parallel plate capacitor and is designed using COMSOL Multiphysics. An actuation displacement of 170 nm was used, which is the required distance to tune the filter over the whole visible range (400–700 nm). There are two designs proposed for the optical filter: the first was optimized to provide maximum transmission and the other is optimized to have minimum full-width-half-maximum (FWHM) value. The first design has a maximum transmission percentage of 94.45% and a minimum transmission of 86.34%; while the minimum FWHM design had an average FWHM value of 7.267 nm. The results showed improvements over the current commercial filters both in transmission and in bandwidth.

## 1. Introduction

Since the development of hyperspectral imaging, the technology has proven to be useful in many applications. Hyperspectral imaging techniques capture a three-dimensional cube containing spectral-spatial information about the scanned object. This cube is sometimes also called the hypercube [1]. The cube is formed by imaging the spatial dimensions at different spectral bands, forming a contiguous spectrum of spatial-spectral information about the scanned object. This captures spectral information that cannot be acquired by a color camera alone [2,3]. Such information enables the detection and classification of material properties throughout its spectral fingerprint [4]. This allows hyperspectral imaging to be utilized in many applications such as medical diagnostics [1,5], food and water quality check [6,7], agriculture crop monitoring [8,9], archaeology [10,11], and astronomy [12,13]. Despite the potential utilities, commercial hyperspectral cameras are expensive and are relatively large. As a result, the spread of hyperspectral imagers is limited [2].

Hyperspectral imaging systems are classified according to their spectral range, acquisition mode, type of detector, and spectral filtering technology [1]. Traditional hyperspectral imagers either scan the spectral bands or the spatial dimension of the hypercube. These methods are relatively slow due to the filters used and the sensitive moving parts required, which adds complexity to the system. The snapshot method, on the other hand, enables the system to capture a full hypercube in one shot. However, there is a compromise in the form of a lower spatial resolution to allow for a better spectral resolution. This is because, in a snapshot hyperspectral camera, pixels consist of a number of sub-pixels, each with a spectral filter applied [2]. This, in turn, increases the pixel size; hence, there is a decrease in the spatial resolution and an increase in the spectral resolution. The key component in any hyperspectral system is the spectral filtering technique utilized [14]. Almost all specifications of the system are dependent on the selected spectral filtering technology. Hyperspectral imaging systems can employ various methods, such as filter wheels [15,16], prisms [17,18], gratings [19], and tunable optical filters [20,21,22,23]. Tunable optical filters offer an acceptable bandwidth and switching time without the compromise of the special resolution. They can be based on different principles of operation, such as acousto-optic tunable filter (AOTF) and liquid-crystal tunable filter (LCTF). Each technology has its own advantages and disadvantages. For example, AOTFs offer a rapid wavelength tunability; however, they suffer from a very low transmission percentage. LCTFs, on the other hand, offer a wide tuning range, but they have low scanning speed along with low transmission percentage [24]. Cutting-edge LCTFs currently available in the market have a maximum transmission that does not exceed 70%, with a full-width-half-maximum (FWHM) of 35 nm. Other available designs are optimized to have a narrow FWHM value of 10 nm; however, this impacts the transmission percentage value, reducing it to less than 50% at its maximum [25].

Fabry-Pérot interferometers demonstrate optical filtering performance, enabling them to be used as tunable filters. Those interferometers consist of two partially reflective mirrors facing each other, separated by a gap. The mirrors’ reflectivity, along with the gap’s thickness and material, define the filtering behavior of the structure. Highly reflective mirrors are required for narrow bandwidth filter, although they must have low attenuation so as to not reduce the filter’s transmission, which renders metallic mirrors not particularly suitable. Multilayer-dielectric mirrors are ideal for that, although they require many bilayers and, therefore many fabrication steps. Employing one thin plasmonic layer with a few dielectric layers can offer a good compromise. Several types of material have been utilized in the separating gap [26]. In order to tune the optical filter, either the gap’s thickness or refractive index (RI) is changed to control the central wavelength of the filter’s transmission band. Varying the gap’s thickness is more practical, because it simply requires an actuator, not a supply of different materials, or special materials of electrically controlled properties to change the RI. Micro-electro-mechanical systems (MEMS) actuators are devices that convert electrical signals into motion, typically within micrometer range. They offer precise and reliable movement, a compact size, and the potential for mass production which in turn lowers the overall price. Electrostatic MEMS actuators utilize the electrostatic force resulting from applying different voltage values to two electrodes placed close to each other [27]. Such actuators are perfect for the design in hand as they offer a nanometer precision which is translated into a high wavelength tunability accuracy for the tunable filter [28]. Electrostatic actuation also does not interfere with the optical functionality of the filter, because the actuator could be designed separated from the filter layers. Such actuators also do not produce heat or parasitic effects that may affect the filter’s operation. Therefore, electrostatic MEMS actuators are suitable method of actuation for such filters.

In this paper, we present electrostatically actuated tunable filters for hyperspectral imaging systems. The optical filters are based on hybrid plasmonic-dielectric thin films. The filters were designed, using MATLAB from The MathWorks, Inc., a MATLAB code based on the transfer matrix method developed in-house, subsequently, the results were verified by electromagnetic wave numerical simulations using COMSOL. The actuator was designed analytically, and then miniaturized versions were simulated electromechanically on COMSOL Multiphysics for verification.

## 2. Device Design

**Figure 1:**
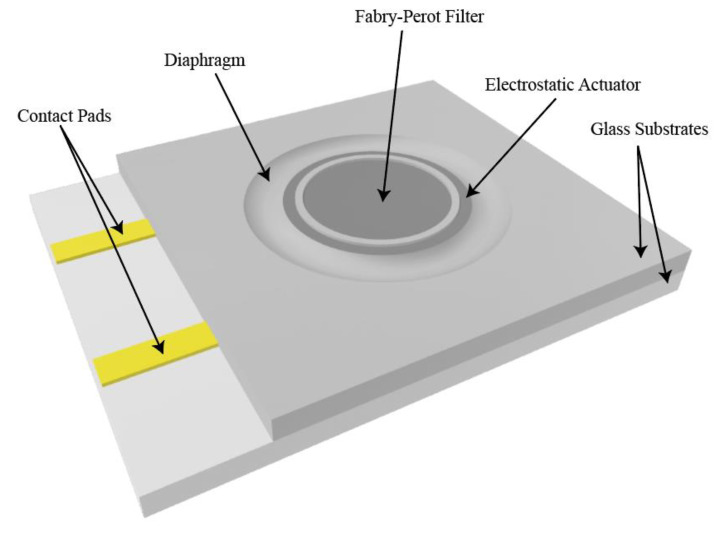
Schematic representation of the MEMS chip.

One of the main advantages of the proposed device that it is a stand-alone chip that can be placed in front of an ordinary monochromatic camera to turn it into a hyperspectral imaging one. Figure 1 shows a schematic of the designed filter. As shown in the figure, the MEMS chip consists of two glass wafers bonded together, holding the circular mirrors forming a Fabry-Pérot filter, along with the actuating electrodes forming an electrostatic parallel plate capacitor. The electrodes are chosen to be from indium tin oxide (ITO) [28], aluminum [29] or silver [30] due to their electric conductivity and adhesion to glass substrate. A diaphragm is realized by wetly etching a circular trench in the upper wafer to reduce its thickness making it easy to move. This forms a suspension system that contains the movable mirror of the filter, and the movable electrode of the actuator. The lower wafer contains the fixed mirror and the fixed electrode. The mirrors are deposited in the middle area of the two wafers on the inner faces to form the filter, while the electrodes are of ring shapes surrounding them. This design allows decoupling of the actuation-controlling electrodes from the filter mirrors; hence, controls the tuning gap is controlled without interfering with the optical filter operation.

### 2.1. Filter Design

In this section, the operation principle of the optical filter is discussed, along with the design criteria for hyperspectral imaging applications. As mentioned earlier, the filter is based on a Fabry-Pérot interferometer and actuated using an electrostatic MEMS actuator. Fabry-Pérot resonators are based on interference between the incident light on it and the light reflected multiple times inside the resonator. The distance between the two reflecting mirrors defines the resonator performance and hence the central wavelength of the Fabry-Pérot filter. The transmission profile of a typical Fabry-Pérot filter is described by the following relation: $d=\lambda \left(2m\pi +\phi_{a}+\phi_{b}\right)/2\pi n_{gap}$

(1) $$T=\left(1-r^{2}\right)/\left(1+r^{2}-2rcos\delta \right)$$

where, r is the mirror’s reflection coefficient, and δ  is the total phase. The maximum transmission occurs when $\delta =\phi_{prop}-\left(\phi_{a}+\phi_{b}\right)=2m\pi$, where $\phi_{prop}=4\pi n_{gap}d$ is the propagation phase shift, $\phi_{a}$ and $\phi_{b}$ are the phase shift due to reflection from the two mirrors respectively, d is the mirror’s gap thickness, and $n_{gap}$ is the gap’s material RI. To determine the cavity thickness required for a certain central wavelength, we apply the previous phase condition, and rearrange to find the cavity thickness; which results in the relation $d=\lambda \left(2m\pi +\phi_{a}+\phi_{b}\right)/2\pi n_{gap}$ [31]. The FWHM of the Fabry-Pérot filter is an important parameter, as it measures how narrow the spectral line of the filter is. For normal incidence, it can be determined by the following equation:

(2) $$FWHM=\left(1-r\right)\lambda^{2}/2\pi \sqrt{r}n_{gap}d$$

For the oblique incidence, the gap thickness is replaced by the effective gap thickness $d_{eff}=d/cos\;\theta$ where θ is the angle of incidence [32]. As shown by the previous equations, the mirror’s reflectivity greatly affects the filtering performance of the interferometer; therefore, the mirrors’ design is an important factor to consider in the design process. Several designs have been presented for mirrors; such as, dielectric mirrors, metallic reflective mirrors and hybrid designs [28,33]. Dielectric materials exhibit low attenuation properties; which enables the addition of a large number of layers without dramatically affecting the transmission percentage which enables achieving narrow bandwidths. These narrow bandwidths are achieved by adding more layers, because adding more layers introduces more restrictions on which frequencies meet the conditions of constructive interference; and hence, sharp band-pass filters with high transmission filters are produced. However, dielectric mirrors require a large number of layers to achieve that narrow bandwidth, which therefore requires many fabrication steps. Beside the fabrication cost resulting from that, it also renders the process more prone to fabrication discrepancies in material properties and in layers’ thicknesses [34,35]. The large number of fabrication steps adds complexity to the filter’s implementation and sets it at a higher price point. Metallic mirrors, on the contrary, exhibit high attenuation due to the absorption nature of the metallic layer used. This results in lower transmission percentage values at the pass band of the filter. However, one metal layer is enough to form a metallic mirror, which removes the complexities involved in the fabrication of the dielectric layer and lowers the cost of the filter. The bandwidth of the metallic mirrors is determined by the nature of the metal used. The range of light the metal reflects is determined by the atomic structure of the material, as described by Drude-Lorentz model. For instance, silver bandwidth covers the entire visible range along with part of the infra-red range. Combining both metallic and dielectric layers in the mirror design allows the filter to have both advantages. Using one thin metal layer-of just few nanometers thickness to not cause high attenuation-along with just few dielectric layers, produces a mirror with high performance in terms of the narrow bandwidth, high transmission percentage and sharp line filter, without requiring many fabrication steps.

**Figure 2:**
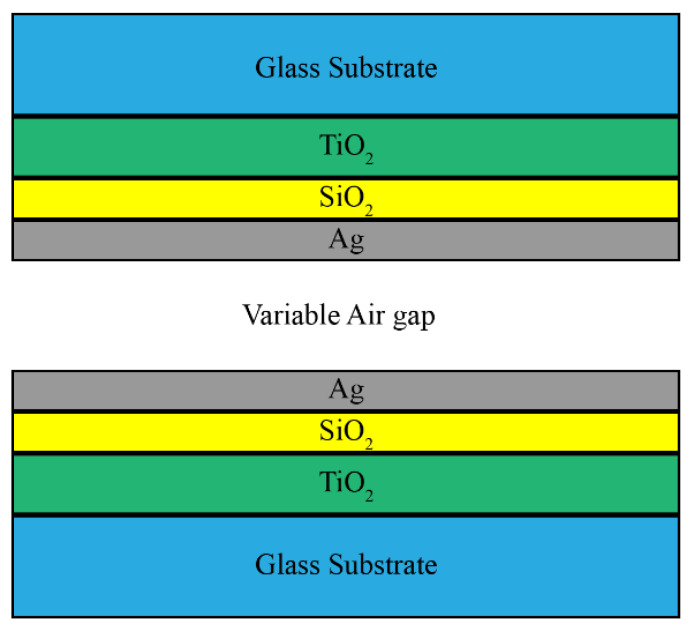
Schematic representation of the filter’s layers.

The filter presented in this paper consists of two partially reflective mirrors facing each other. Each mirror is a glass substrate with hybrid dielectric-plasmonic thin films deposited on it, as shown in Figure 2. Only three layers for each mirror are sufficient: two dielectric layers, and one thin metallic layer. This design is chosen to have both dielectric layers and metallic layers deposited on each other to add more degrees of freedom in the design process, and compensate for the draw backs of the metallic mirrors. All the materials chosen in this design have extremely low extinction coefficients in the visible range to prevent attenuation due to material absorption, along with good adhesion to each other and to the substrate. For the dielectric layers, two materials are chosen: one has to have a high RI, while the other has to have a low RI. For applications in the visible light range, silicon dioxide (SiO_2_) and titanium dioxide (TiO_2_) are commonly used [35]. Both materials have a nearly zero extinction coefficient in the visible range (400–700 nm), and have a sufficiently different refractive index values: TiO_2_ is the high-RI material that has an average value of 2.17, according to Sarkar et al. [36], and SiO_2_ is the low-RI material with a value of around 1.46 according to Malitson [37]. For both materials, the RI dependence on the wavelength is taken into consideration in the model. In the visible range, silver (Ag) has a low extinction coefficient; therefore, it has a low absorption in this band, which translates to a high transmission in the filter performance. In addition, silver has good adhesion to the used materials [35]. Therefore, the metallic layer in this design is chosen to be silver. The filter is symmetrical: both mirrors have the same number of layers, and each layer in each mirror has the same material and thickness.

The thickness of the layers defines the filter’s performance. Hyperspectral imaging requires the optical filter to reach the maximum transmission possible in order to harvest the maximum power from the specified spectral band. Furthermore, the spectral band needs to be as well-defined as possible. Hence, the bandwidth needs to be as narrow as possible, in order to enable the separation between a higher number of bands, which increases the spectral resolution. The rejection band must cover the whole operation band of the filter and the camera sensor used. The presented structure has some degrees of freedom in the design; therefore, we will present two designs for tunable hyperspectral imaging filters: one is optimized for maximum transmission, and the other is optimized for minimum FWHM. Optimization is performed using a MATLAB code developed in-house and based on the transfer matrix method, then verified by electromagnetic wave numerical simulations by COMSOL. The resulting values of the layer thicknesses are summarized in Table 1.

The deposition of thin films initiates intrinsic and thermal stresses in the wafer due to lattice mismatch the difference in thermal coefficient. Hence, such effects must be considered in the design process. Deposition of thin film of TiO_2_ on glass substrate using electron beam evaporation causes tensile residual stress in the order of 170 MPa [38]. While deposition of thin film of SiO_2_ on a thin film of TiO_2_ causes a compression stress in the same order to form [39,40]. Deposition of silver on the other hand causes a tensile stress in the order of 18 Pa which gives a radius of curvature in the range of thousands of kilometers according to Stoney’s equation [38]. Therefore, carefully designing the stresses in the films during the fabrication process will result in an almost perfect plan filter.

Fabry-Pérot filters are known to be dependent on the angle of incidence; therefore, for real life imaging applications will dictate the filter to be inserted in an optical system that allow rays collection, and collimate them on the filter. Such systems have been presented earlier in the literature [40]. Some systems allow for a field of view of 30 degrees [41].

### 2.2. Actuator Design

**Figure 3:**
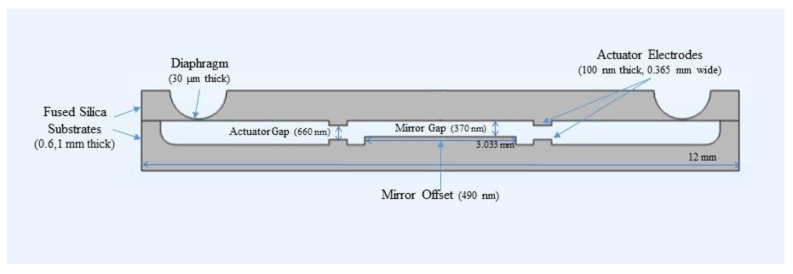
Cross sectional view of the MEMS actuated filter, with the dimensions indicated.

The tunable air gap in the filter is controlled by an electrostatic actuator. As indicated by the name, the actuator uses electrostatic force to bend the diaphragm and thus move the upper mirror closer to the bottom mirror. The actuator consists of two parallel plate electrodes, which are perfect suited for Fabry-Pérot filter structures because they have the same simple structure. As shown in Figure 3, the actuator is added as a pair of parallel plates around the mirrors. For an electrostatic parallel plate actuator, at displacement value x, the driving voltage is given by the following relation:

(3) $$V=\sqrt{2kx{\left(g_{act}-x\right)}^{2}/\varepsilon A_{act}}$$

where k is the diaphragm spring constant, $g_{act}$ is the initial gap of the actuator (at zero applied voltage), ε is the permittivity of the material between the two electrodes and $A_{act}$ is the actuator electrode area. The actuator is controlled by applying a DC voltage across the two electrodes. The actuator is allowed to move by only one-third of the initial gap. If the voltage is increased to exceed this distance, the movable plate may stick to the fixed one and become immovable. This phenomenon is known as pull-in, and the voltage limit is called the pull-in voltage [28].

As indicated by the dimensions in Figure 3, the filter is designed to be in the millimeter scale, and it should be operated at appropriate voltages. The upper substrate has a thickness of 0.6 mm and a diameter of 12 mm, while the lower substrate has the same diameter but a thickness of 1 mm. The mirror diameter is determined to be 3.033 mm and the electrodes’ width and the initial gap between them is 0.365 mm and 660 nm respectively for acceptable voltage value of actuation. The diaphragm has a minimal thickness of 30 µm, but its thickness increases due to the circular nature of the trench wetly etched in the wafer. The design is initially determined by the previous analytical formula, then it is to be optimized using COMSOL Multiphysics. However, simulating the actual design by linked mechanical and electrostatic modules in the millimeter scale is not successful due to the computational power needed. Therefore, the design is scaled down by factors from 1/1000 to 16/1000. All the design dimensions are changed accordingly except the mirrors gap and the actuator gap to keep the filter functionality intact. Only the mechanical simulation could be done with real dimensions, which is important to get the diaphragm spring constant. As the structure is circularly symmetric, only one-quarter of it can be simulated and symmetry plans can be put to depict the complete circular shape. This helps saving the computational power and time. The cross section of the structure –depicted in Figure 3 is drawn in 2D, then revolved by 90° to realize one-quarter of the 3D structure. For all the simulations, normal automatic mesh is applied, and stationary study is used. The boundary condition used is applying a fixed constraint on the two substrates perimeters and on the below surface of the lower substrate. For the electromagnetic simulation, the actuation voltage and ground boundary conditions are applied on the electrodes. While in the mechanical simulation done in the real dimensions to get the spring constant of the diaphragm, a force is applied to its center equivalent to the expected electrostatic force between the electrodes if they could be simulated in these large dimensions. This applied force F moves the movable structure downwards by a certain distance x, then Hooke’s law (F=−kx) is used to get the stiffness k. A graph is plotted between the applied force and displacement, then the spring constant is obtained as the negative of the slope of the obtained straight line.

## 3. Results and Discussion

For evaluating the filter optical performance, the transmission curves are generated using the transfer matrix, method then verified using the COMSOL electromagnetic waves module. Normal incidence is assumed for all filter curves, and the transmission range of interest is the visible wavelength range. Based on the transmission curves generated, the required parameters for evaluating a filter for usability in hyperspectral imaging applications are obtained. These parameters are: the maximum transmission percentage, FWHM of the spectral pass band, the change in FWHM with the wavelength, and the linearity of the scanned wavelength with the tuned gap. For the actuator, the main design parameters obtained were the tuning range, diaphragm stiffness, and maximum and minimum required voltage for actuation. As mentioned earlier, a model that describes the actuator with scaled-down dimensions are developed, so all the previous parameters changes are studied by considering the scaling factor.

### 3.1. Filter Design Results

**Figure 4:**
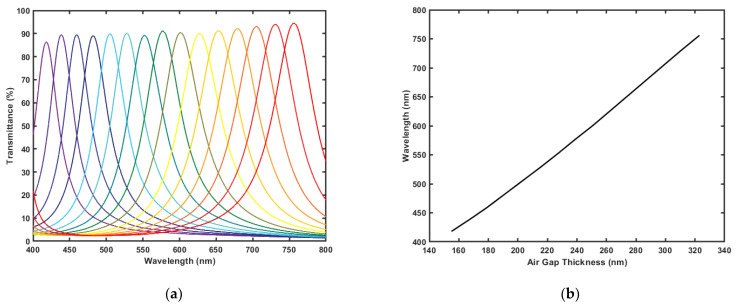
Results generated by MATLAB code for the design optimized for maximum transmission: (**a**) Transmission spectra of the filter at different air gap values; (**b**) Resonance wavelength versus the tuned air gap thickness of the filter.

As mentioned earlier, two designs are presented: one is optimized to have as maximum transmission as possible with rather acceptable FWHM values, and the other is optimized for the minimum FWHM values possible with acceptable transmission values. The acceptable range for each value is determined from the available filter specifications in the market [25]. Figure 4a shows the transmission spectra of the design optimized for maximum transmission. As shown, the filter exhibits the highest transmission at the longest wavelength in the band with a value of 94.45%, and a minimum transmission at the violet color with value of 86.34%. The FWHM of this design is rather quite high for hyperspectral filters, it has an average FWHM value of 56.933 nm. Both the transmission percentage and the FWHM increase as the wavelength increases. The change of the FWHM values over the entire tunability gap is 27 nm; it starts at 38 nm at 417 nm then it gradually increases to reach its maximum at 758 nm with a value of 56 nm. Figure 4b shows the change of the peak wavelength with tuning the air gap. As shown, the resonance wavelength changes linearly with the gap value.

Filters based on Fabry-Perot interferometers exhibit a secondary peak behavior. The spectral distance between the two peaks is determined by the mirrors’ reflectivity and the gap values. The filter is designed to have one peak in the spectral range from 400–700 nm. The range could be extended to 400–750 nm; however, a secondary peak shows up at 400 nm when tuning the filter to 750 nm. Such a parasitic peak has a signal to noise ratio of 5.278 for the maximum transmission design in Figure 4a. If the application does not allow for such noise, a blocking filter must be used to ensure effective spectral filtering.

**Figure 5:**
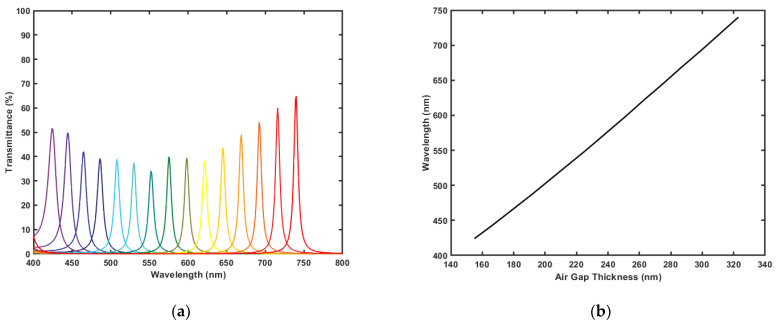
Results generated by MATLAB code for the design optimized for minimum FWHM: (**a**) Transmission spectra of the filter at different air gap values; (**b**) Resonance wavelength versus the tuned air gap thickness of the filter.

For the design optimized to achieve the sharpest resonance peaks, the transmission spectra are shown in Figure 5a. The filter covers the whole visible range from 400 nm up to 750 nm. The transmission behavior of this filter is not linear. It has a minimum value at the 552 nm wavelength with transmission value of 34.14%, and a maximum transmission value at the red color with transmission value of 64.95%. The FWHM values have an average of 7.267 nm, which is lower than the narrow bandwidth filters offered in the market [25,42]. Like the maximum transmission filter, tuning the air gap thickness changes the resonance wavelength linearly as shown in Figure 5b.

### 3.2. Filter Results Verification

To verify the results generated in the design process by the MATLAB code, the filter structure is studied by COMSOL numerical simulations under the same illumination and incidence conditions. The Electromagnetic Waves, Frequency Domain interface, found under the Wave Optics module, is used in this study. This interface uses Helmholtz equations to solve for the time-harmonic electromagnetic field distributions [43]. Two-dimensional model is used, which solves in the modeled two-dimensional structure and assumes that the third dimension is infinite. To save computational time and power, the width of the device is chosen to be 10 µm, which is large enough to not function as a waveguide and small enough to avoid meshing problems. The thicknesses of the filter layers are as in Table 1. However, substrates thicknesses are set to be large enough not to function as layers and small enough not to cause meshing and computational issues, which is 300 nm. Materials are set to be matching the one used in MATLAB code in the design process. This simulation could be enhanced to match the actual device by increasing the substrate thicknesses and the width value, and extend it into three-dimensional model; however, the simulation performed and parameters chosen matched the analytical and transfer matrix calculated results, which indicated that they are still sufficient to describe the real device behavior.

**Figure 6:**
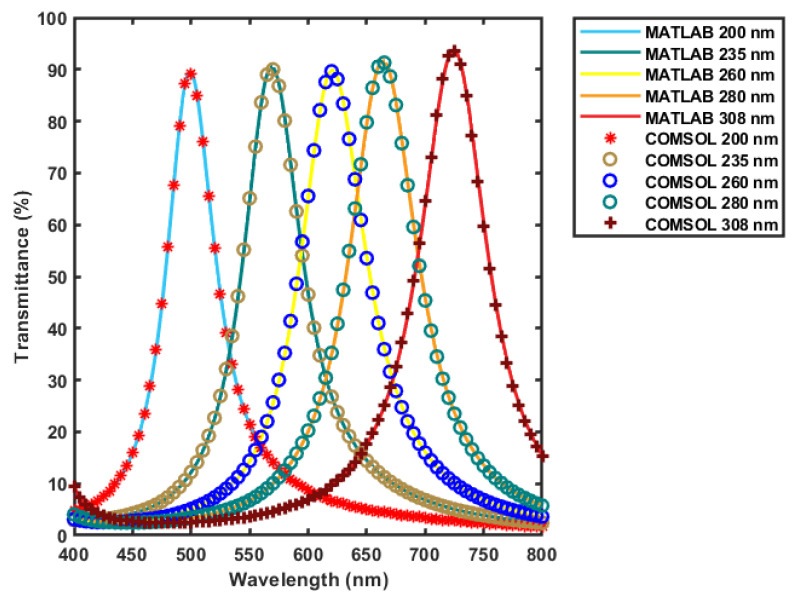
Comparing the transmission spectra of the filter optimized for maximum transmission at different gap values obtained from COMSOL and MATLAB.

**Figure 7:**
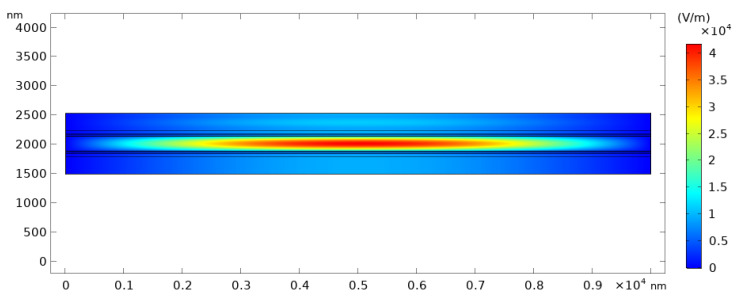
Electric field distribution in the filter with gap value of 260 nm at resonance wavelength of 625 nm.

Figure 6 shows the results obtained from the two-dimensional model in COMSOL compared to the results generated by the MATLAB code used in the design process. All the spectrum has been simulated and compared; however, five curves only was chosen to display. Results are matching each other, with a maximum error of 2.32% at gap value of 308 nm, and an average error of 0.7302% from all the spectra of the different gap values; which proves the reliability of the results achieved by the MATLAB code. As illustrated in Figure 7, at the resonance wavelength, the electric field resonates inside the cavity of the filter and builds up to have maximum value.

**Figure 8:**
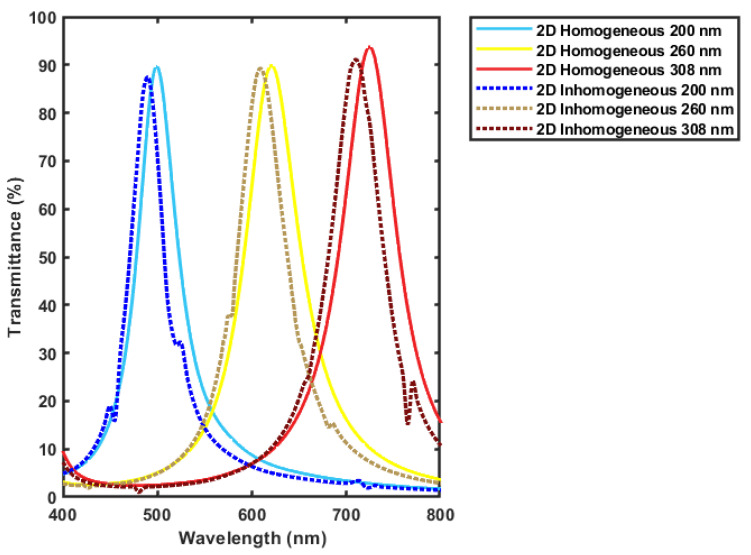
Comparing the transmission spectra of the filter optimized for maximum transmission with and without lateral in homogeneity in the layers’ thicknesses.

The filter is also simulated with the introduction of lateral inhomogeneity in the thicknesses of the deposited films. Assuming a change of 10% in each layer, the worst cases is where the change of 10% of all the layer’s thickness occur [35]. This case is simulated and compared to the homogeneous design results. The inhomogeneity is assumed to be a liner change across the width of the filter to be at its minimum at the middle and its maximum at the edges. As shown in Figure 8, a few spikes that does not disturb the general peak shape are found as an effect of the layer’s inhomogeneity, and the central wavelength is shifted to a smaller wavelengths’ values. For the simulated values the maximum error is around 1.9%. This shift could be treated by proper calibration of the fabricated filter.

### 3.3. Actuator Design Results

As seen in the optical design, the actuator should move the mirror from a gap of 150 nm to a gap of 320 nm. This is could be achieved by applying certain minimum and maximum voltages on the actuator plate. These values are needed to be determined, along with diaphragm stiffness. The structure is designed to have dimensions at the millimeter scale as described in Section 2.2. Therefore, simulating the whole structure at the millimeter scale is not possible due to the computational power required. To overcome this issue, the design is scaled down to the micrometer scale, as mentioned earlier, with down-scaling factors. Then results are extrapolated to the original design scale. Only the mechanical performance could be verified by COMSOL at the millimeter scale to determine the actual value of the diaphragm stiffness k. The voltage required for actuation, on the other hand, is not possible to be calculated using COMSOL at this scale, so it is calculated analytically and compared to the extrapolated model.

**Figure 9:**
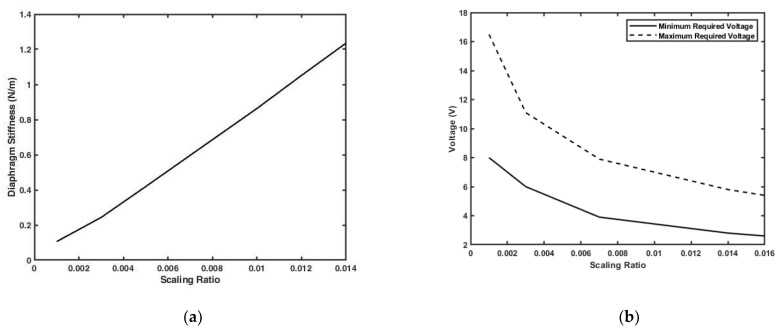
Actuator performance at different scaling factors obtained by COMSOL: (**a**) Diaphragm stiffness at different scaling factors.; (**b**) Minimum and maximum required actuation voltage at different scaling ratios.

Using this approach, the relation between the scaling factor X and the diaphragm stiffness k is found to be linear, as shown in Figure 9a, so it is safe to fit it with a first order polynomial equation, which is found to be:

(4) Diaphragm Stiffness=88 X−0.00832

where, X is the scaling down factor. The minimum and maximum voltages required for actuation verses the scaling down factor are as shown in Figure 9b. As illustrated, the relation is not quite linear, therefore they are fitted with the following formulas

(5) $$Minimum\;Actuation\;Voltage=7.6\;{\left(700X\right)}^{-0.48}$$

(6) $$Maximum\;Actuation\;Voltage=15.7\;{\left(700X\right)}^{-0.527}$$

**Figure 10:**
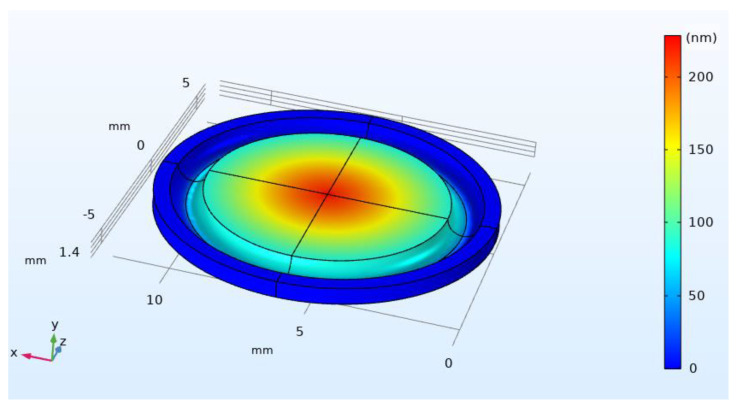
Upper substrate displacement at the original design scale (scaling factor 1) in the mechanical model.

To validate the model, the diaphragm stiffness is calculated at the millimeter scale (X value of 1) using COMSOL mechanical model, and compared with the predicted value from the extrapolated formulas. The predicted value from the extrapolated data is 88 N/m and the calculated value using COMSOL is 87.48 N/m, Figure 10 shows the mechanical simulation results from COMSOL mechanical module. The minimum and maximum voltages for actuation are calculated analytically using equation 3 and from the extrapolated formulas. The analytically calculated results are 0.308 V and 0.466 V for the minimum and maximum voltages respectively; while the extrapolated results are 0.327 V as the minimum voltage and 0.497 V as the maximum voltage. Indeed, these values are a little different from those obtained analytically, but this is expected due to the fringes capacitance that is not accounted in the analytical model. The stated empirical formulas might also be useful for the predication of an intermediate design values if needed.

## 4. Conclusions

A tunable optical filter has been designed and optimized for hyperspectral imaging applications. The presented filter is based on Fabry-Pérot interferometer, utilizing hybrid plasmonic-dielectric thin film mirrors, and actuated using an electrostatic MEMS actuator. Two optical filter designs have been illustrated: one was optimized to have maximum transmission, and the other was optimized to have a minimum FWHM value. The filter optimized for maximum transmission, presented transmission rates up to 94.45% with and average FWHM value of 56.933 nm; whereas, the filter optimized for a minimum FWHM value offers a transmission up to 64.95% with an average FWHM value of 7.267 nm. These vales, to the best of our knowledge, exceeds the designs available in the market. The designs were attained by modeling the multilayers by the transfer matrix method, and verified by numerical modeling. Both methods demonstrated similar performance, with error less than 2.32%. The actuator was designed and verified by analytical modeling and by extrapolating the values from scaled-down COMSOL models. It offered an actuation range of 170 nm, which enabled tuning of the filter’s gap to scan the entire visible range (400–700 nm) by varying the applied voltage between 0.327 and 0.497 V. The design could be scaled up or down based on the desired application. The fabrication process has been started by the thin films’ deposition; then, it will be followed by the complete structure fabrication and characterization.

## Figures and Tables

**Table 1:** Optimized layers’ thicknesses for filters structure targeting maximum transmission and minimum FWHM.

Layer	Maximum Transmission Filter	Minimum FWHM Filter
TiO_2_ Layer	43 nm	63 nm
SiO_2_ Layer	25 nm	50 nm
Ag Layer	20 nm	40 nm
Variable Air Gap	(150 nm–320 nm)	(150 nm–320 nm)

## Data Availability

Not applicable.
